# Post-cardiac Arrest Ventilator Triggering

**DOI:** 10.5005/jp-journals-10071-23155

**Published:** 2019-04

**Authors:** Anant Vikram Pachisia, Sandip Bhattacharyya

**Affiliations:** 1,2 Department of Critical Care Medicine, Asian Institute of Medical Sciences, Faridabad, Haryana, India.

## Abstract

**How to cite this article:** Pachisia AV, Bhattacharyya S. Post-cardiac Arrest Ventilator Triggering. Indian J Crit Care Med 2019;23(4):196.

Sir,

A patient, diagnosed as *Aspergillus* pneumonia was referred to our hospital. On evaluation, he was found to be hypoxic, chest X-ray revealed large left-sided pneumothorax with empyema. Orotracheal intubation was done. Chest tube was inserted and negative suction was applied. There was bronchopleural fistula because of which there was large air leak (~200 mL). Eventually, the patient had cardiac arrest after couple of days in ICU. CPR was done as per ACLS protocol but the patient could not be revived. Before turning off the ventilator we realized that on volume control mode, respiratory rate was set at 15/min but delivered breath rate was almost 30/min. To check for any error, we reduced the respiratory rate to 1/min, but still delivered respiratory rate was 29–30/min with tidal volume varying from 200 mL to 300 mL (Fig. 1). We ultimately realized that this was due to large bronchopleural fistula with negative suctioning causing the ventilator triggering. We were only able to resolve this after clamping the chest tube (Fig. 2).

**Fig. 1:**
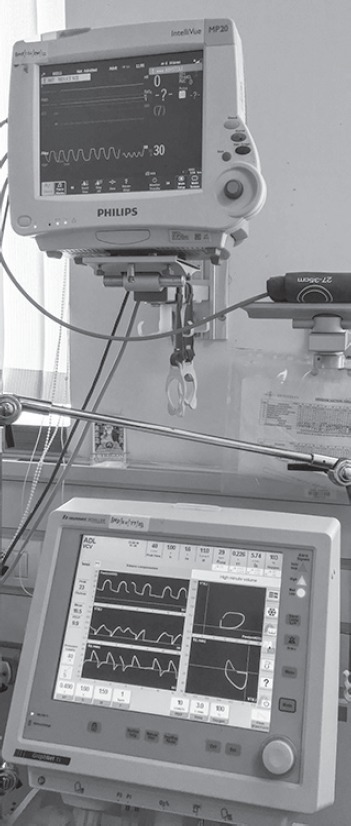
Ventilator triggering post-cardiac arrest

**Fig. 2:**
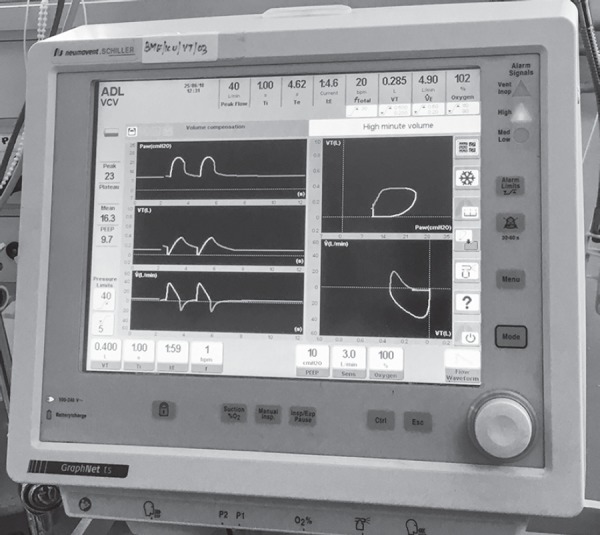
Post-chest tube clamping ventilator triggering stopped

Kaloria et al. and Arbour R have reported ventilator triggering in brain-dead patients caused by cardiac oscillations.^1,2^ Sager et al. in their case report have discussed about ventilator triggering in a patient with tubercular bronchopleural fistula. They were able to reduce the leak from bronchopleural fistula by decreasing the trigger sensitivity or by decreasing the degree of chest tube suction.^3^ The mechanism for air leak in our patient post-cardiac arrest was that air leak from the distal airway lowers proximal airway pressure below the set trigger sensitivity and thus triggers inspiration, without patient effort. We report this case because the constant ventilator triggering caused by air leak from bronchopleural fistula post-cardiac arrest can perplex the clinician and should be kept in mind when dealing with air leak in chest tube.
